# Sleep

**Published:** 1883-07

**Authors:** 


### SLEEP.

MEN, women and children require just so much sleep, and if they do not have it, suffer in consequence. I do not think a person should be waked in the morning, and for this reason when a man falls asleep he is in the shop for repairs, as the railroad men say. His frame and all its intricate machinery is being overhauled and made ready for the next day's work. The wear of the previous day is being repaired. Nature is doing that herself. She knows what the tired frame needs just as she knows how to make the heart throb and send the blood coursing through the veins. Then she takes that tired frame, lays it down on a bed, surrounds it with the refreshing air of night, covers it with the soft darkness and lets the man rest. "Tired nature's sweet restorer, balmy sleep," visits him, and as the hours wear by his energies are renewed, his strength comes back, and finally, when morning breaks and the sunlight steals through the lattice, he opens his eyes and is himself again. Or if he is early to bed he awakes with the cocks crowing. Now who shall go to that man's side an hour before he opens his eyes and say to nature, stand aside and let him get up ; he has had enough of rest ? Well, nature will say : "You can take him if you will, but I will charge him with an hour's loss of sleep and I'll collect it out of his bones and nerves and his hair and eyesight. You can't cheat me. I'll find property to levy on!"

FAME is not such a very sure thing. Tom Marshall' last words, as he turned his face toward a window were : "This is the end! I am dying on a borrowed bed, under a borrowed blanket, in a house built by public charity! Bury me under that oak tree, where there is plenty of room ; I have been crowded all my life."

